# A comparison of the effects of lung protective ventilation and conventional ventilation on the occurrence of atelectasis during laparoscopic surgery in young infants: a randomized controlled trial

**DOI:** 10.3389/fmed.2024.1486236

**Published:** 2024-10-10

**Authors:** Kun Yue, Jingru Wang, Huangxing Wu, Yingying Sun, Yin Xia, Qi Chen

**Affiliations:** ^1^Department of Anesthesiology and Perioperative Medicine, The Second Affiliated Hospital of Anhui Medical University, Hefei, Anhui, China; ^2^Department of Anesthesiology and Perioperative Medicine, Anhui Provincial Children's Hospital, Hefei, Anhui, China

**Keywords:** pulmonary atelectasis, lung protective ventilation, positive end-expiratory pressure, recruitment maneuvers, infant, laparoscopy, ultrasonography

## Abstract

**Objective:**

This study utilized lung ultrasound to investigate whether lung protective ventilation reduces pulmonary atelectasis and improves intraoperative oxygenation in infants undergoing laparoscopic surgery.

**Methods:**

Eighty young infants (aged 1–6 months) who received general anesthesia for more than 2 h during laparoscopic surgery were randomized into the lung protective ventilation group (LPV group) and the conventional ventilation group (control group). The LPV group received mechanical ventilation starting at 6 mL/kg tidal volume, 5 cmH_2_O PEEP, 60% inspired oxygen fraction, and half-hourly alveolar recruitment maneuvers. Control group ventilation began with 8–10 mL/kg tidal volume, 0 cmH_2_O PEEP, and 60% inspired oxygen fraction. Lung ultrasound was conducted five times—T1 (5 min post-intubation), T2 (5 min post-pneumoperitoneum), T3 (at the end of surgery), T4 (post-extubation), and T5 (prior to discharge from the PACU)—for each infant. Simultaneous arterial blood gas analysis was performed at T1, T2, T3, and T4.

**Results:**

Statistically significant differences were observed in pulmonary atelectasis incidence, lung ultrasound scores, and the PaO_2_, PaCO_2_, PaO_2_/FiO_2_ ratios at T2, T3, and T4. However, at T5, no statistically significant differences were noted in terms of lung ultrasound scores (4.30 ± 1.87 vs. 5.00 ± 2.43, 95% CI: −1.67 to 0.27, *p* = 0.153) or the incidence of pulmonary atelectasis (32.5% vs. 47.5%, *p* = 0.171).

**Conclusion:**

In infants aged 1–6 months, lung protective ventilation during laparoscopy under general anesthesia significantly reduced the incidence of pulmonary atelectasis and enhanced intraoperative oxygenation and dynamic lung compliance compared to conventional ventilation. However, these benefits did not persist; no differences were observed in lung ultrasound scores or the incidence of pulmonary atelectasis at PACU discharge.

**Clinical trial registration:**

http://www.chictr.org.cn/, identifier: ChiCTR2200058653.

## 1 Introduction

Laparoscopic surgery in infants and toddlers is becoming more widespread because of its minimal invasiveness. During laparoscopic surgery, an increase in intra-abdominal pressure elevates the diaphragm and reduces both chest wall compliance and functional residual capacity (FRC), leading to further atelectasis formation in the dependent lung bases (1). Pulmonary atelectasis contributes to perioperative lung dysfunction and potential injury (2). Neonates, infants and small children have low functional residual capacity, high pulmonary closing capacity and high oxygen consumption, making them particularly susceptible to atelectasis and hypoxemia during laparoscopic procedures (2, 3). Studies have shown that lung protective ventilation (LPV), such as small tidal volumes (4), positive end-expiratory pressure (PEEP) (5, 6) combined with lung recruitment maneuvers (RMs) (7) can be effective in preventing atelectasis in children. However, the LPV remains a subject of debate and is not well studied (8), particularly in infants and neonates. Lung ultrasound (LUS) is a reliable and accurate noninvasive imaging technique that is effective for detecting anesthesia-induced atelectasis in children (9). Considering the potential benefits of lung protective ventilation in adult and pediatrics patients and the limited research on its use in laparoscopic surgery for infants, a randomized controlled trial was conducted to compare lung protective ventilation with conventional ventilation in these surgeries. The aim of this study was to evaluate the effectiveness of lung protective ventilation (LPV) in reducing the incidence of pulmonary atelectasis and improving oxygenation and dynamic lung compliance during surgery in young infants, as assessed by ultrasound, compared to conventional ventilation. We hypothesized that LPV would lead to lower pulmonary atelectasis and higher intraoperative oxygenation and dynamic lung compliance. Additionally, we anticipated that LPV would reduce the risk of postoperative pulmonary complications in young infants.

## 2 Methods

### 2.1 Ethical approval

This study was approved by the Medical Ethics Committee of Anhui Provincial Children’s Hospital, China, on March 23, 2022 (approval number: EYLL-2022-026) and was registered at http://www.chictr.org.cn/ (trialnumber: ChiCTR2200058653; April 13, 2022). For infants in this study, voluntary informed consent was obtained and signed by their parents or legal guardians. This single-center prospective randomized controlled trial was conducted from April 2022 to December 2023 at Anhui Provincial Children’s Hospital in China.

### 2.2 Participants

Infants aged 1–6 months, classified as American Society of Anesthesiologists (ASA) physical status I-II, and scheduled for laparoscopic abdominal surgery under general anesthesia (>2 h) between April 2022 and December 2023 at Anhui Provincial Children’s Hospital were recruited for this study.

The exclusion criteria were those who were preterm infants, had recent pulmonary inflammation (within one month), had preoperative conditions increasing the risks of severe infections, sepsis, or regurgitation and aspiration, or contraindications to radial artery cannulation, classified as ASA III or higher, lacked family consent, or were needed to taken to the postoperative intensive care unit (ICU) after surgery.

### 2.3 Randomization

Patients were randomized via computer-generated sequence, and their group assignment was sealed in envelopes. Patients were divided into two groups: the lung protective ventilation group (LPV group: small tidal volume, positive end-expiratory pressure, and recruitment maneuvers) and the conventional ventilation group (control group: zero PEEP and no recruitment maneuvers) at a 1:1 ratio. A single investigator opened the envelopes and implemented the respective mechanical ventilation protocols. The assessor responsible for conducting the lung ultrasound evaluations was blinded to the group assignments.

### 2.4 Anesthesia protocols and ventilator settings

An intravenous cannula was placed prior to transferring the infant to the operating room. Upon the patient’s arrival in the operating room, routine monitoring of blood pressure (BP), electrocardiogram (ECG), oxygen saturation (SpO_2_), and body temperature was initiated. Following anesthesia induction, invasive arterial pressure monitoring was commenced. Anesthesia induction involved a sequential intravenous slow injection protocol comprising midazolam (0.05 mg/kg), sufentanil (0.3 μg/kg), propofol (3 mg/kg), and cisatracurium (0.1 mg/kg). Following this, a 3.5–4.5 uncuffed endotracheal tube (10) was inserted, and pressure-controlled ventilation was initiated after successful intubation. Prior to intubation, all patients underwent preoxygenation with 60% (11) oxygen. Anesthesia maintenance involved administering a single caudal block of 0.25% ropivacaine (0.6 mL/kg) combined with an intravenous infusion of remifentanil (0.1 to 0.3 μg/kg/min) and inhalation of sevoflurane (2 to 3%) during surgery. The pneumoperitoneum pressure was consistently maintained at 5–7 mmHg.

Throughout the surgery, haemodynamic stability was maintained, and vasopressors were used as needed. Lactated Ringer’s solution was administered at a rate of 10–15 mL/kg/h during surgery. Additionally, suspended red blood cells, plasma, and albumin were administered as needed. At the end of surgery, the administration of sevoflurane via inhalation and the infusion of remifentanil were ceased. In the LPV group, during surgery, the following ventilation settings were used: tidal volume (TV) of 6 mL/kg, respiratory rate (RR) of 24–28 breaths/min, inspiratory/expiratory (I: E) ratio of 1:1.5, PEEP of 5 cmH_2_O, fractional inspired oxygen tension (FiO_2_) of 60% (12) and a flow rate of 2 L/min, with a maximum pressure limit of 30 cmH_2_O. Lung recruitment maneuvers were performed every 30 min (13). Blood pressure and respiratory parameters were assessed prior to lung recruitment maneuvers to ensure the child was ready for the procedure (14). Recruitment maneuvers were performed, in pressure-controlled mode, with a constant driving pressure of 15 cmH_2_O. PEEP was increased in steps of 5 cmH_2_O, from 5 to 15 cmH_2_O, every three breaths. The target recruitment pressure of 30 cmH_2_O was maintained for 10 breaths (15). For the control group, a tidal volume (TV) of 8–10 mL/kg, a respiratory rate (RR) of 24–28 breaths/min, an I:E ratio of 1:1.5, a PEEP of 0 cmH_2_O (16), a FiO_2_ of 60%, and a flow rate of 2 L/min were used, with a maximum pressure limit of 30 cmH_2_O, and lung recruitment maneuvers were not performed. When mechanical ventilation was performed after induction in both groups, the presence or absence of autoPEEP (17) was observed and recorded. Postoperatively, upon awakening from anesthesia, 0.02 mg/kg atropine and 0.05 mg/kg neostigmine were administered after the infant recovered spontaneous respiration (18). The tracheal tube was removed upon observing indications like a conjugate gaze, purposeful movements, the eye opening, a tidal volume > 5 mL/kg, and facial ghosting (19). All infants were transferred to the postanesthesia care unit (PACU) and received supplemental oxygen at a rate of 2 L/min through a simple mask equipped with an oxygen reservoir. Gradually reduced to room air when the SpO_2_ level exceeded 95% and remained stable. After meeting the criteria for discharge from the PACU (20), the infants were returned to their wards.

### 2.5 Lung ultrasound

**Figure 1:**
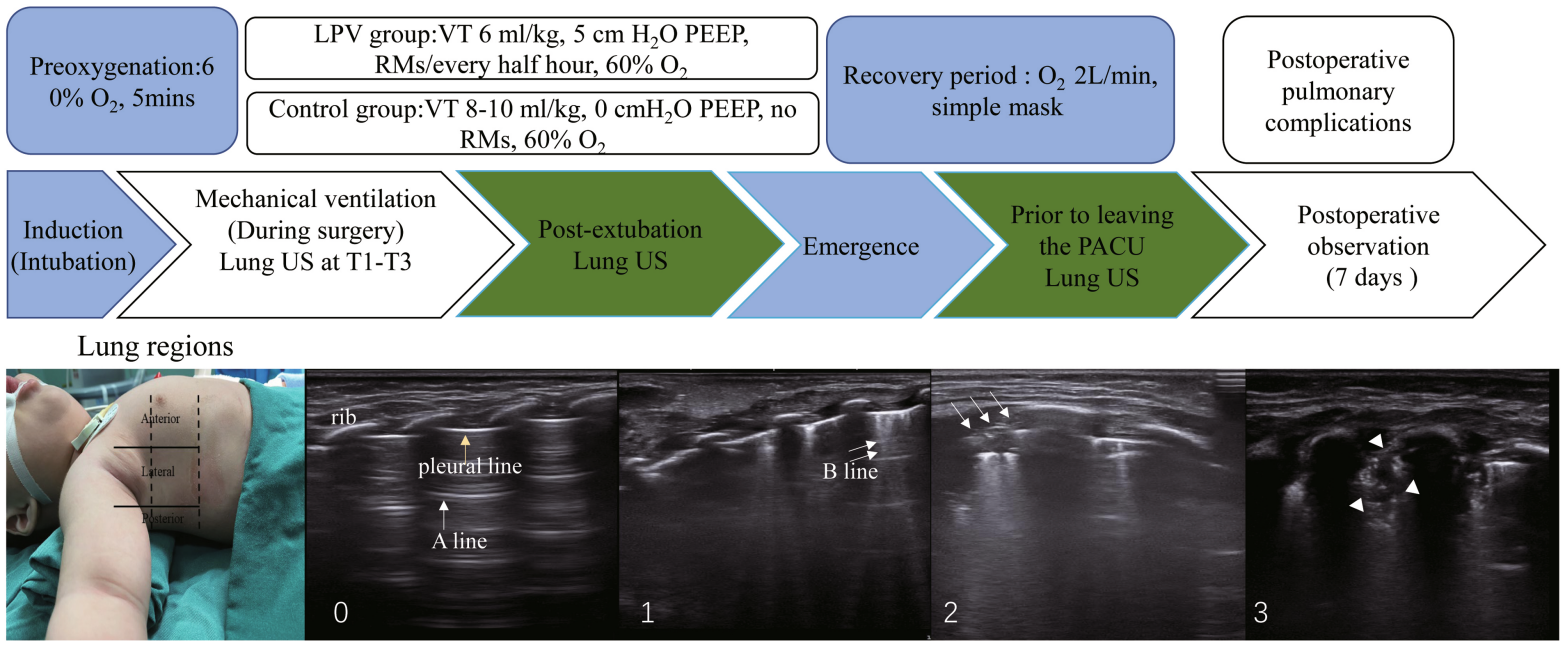
The study protocol and ultrasound lung examination region is shown. From left to right are scores: 0, 0 to 2 B lines; 1, at least three B lines or one or multiple small subpleural consolidations separated by a normal pleural line; 2, multiple coalescent B lines or multiple small subpleural consolidations separated by a thickened or irregular pleural line; 3, consolidation or small subpleural consolidation of more than 1 cm x 2 cm. One yellow arrow, pleural line; one white arrow, A line; two white arrows, B line; three white arrows, subpleural consolidations; white arrowheads, consolidation. Significant atelectasis is determined if any region had a consolidation score of 2. RMs, recruitment maneuvers, PEEP, positive end-expiratory pressure. T1, 5 min post-intubation, T2, 5 min post-pneumoperitoneum, T3, at the end of surgery.

Patients were placed in the supine position and scanned using ultrasound (SONIMAGE HS2-KONICA MINOLTA, Shanghai, China) according to the lung ultrasound score examination method described by Acosta et al. (9, 21). Each half of the chest was divided into three regions (anterior, posterior, and lateral) by the anterior and posterior axillary lines and further divided into six regions by an axial line 1 cm above the nipples. The 12 regions in the lung were scanned sequentially from right to left, from cranial to caudal and anterior to posterior ends. Lung ultrasound scores were assessed using ultrasound at T1 (5 min post-intubation), T2 (5 min post-pneumoperitoneum), T3 (at the end of surgery), T4 (post-extubation), and T5 (prior to discharge from the PACU). The 12 quadrants were assigned a score of 0 to 3 based on the following scoring system: 0, 0 to 2 B lines; 1, at least three B lines or one or multiple small subpleural consolidations separated by a normal pleural line; 2, multiple coalescent B lines or multiple small subpleural consolidations separated by a thickened or irregular pleural line; and 3, consolidation or small subpleural consolidation of more than 1 cm × 2 cm (Figure 1). The consolidation scores were recorded at each time point. Significant atelectasis was determined if any region had a consolidation score of 2 (22). The lung ultrasound score (0–36) was then calculated by adding up the 12 individual quadrant scores, with higher scores indicating more severe aeration loss.

### 2.6 Primary outcome

Lung ultrasound scores and significant atelectasis incidence rates were assessed at T1 (5 min post-intubation), T2 (5 min post-pneumoperitoneum), T3 (at the end of surgery), T4 (post-extubation), and T5 (prior to discharge from the PACU).

### 2.7 Secondary outcomes

**Figure 2:**
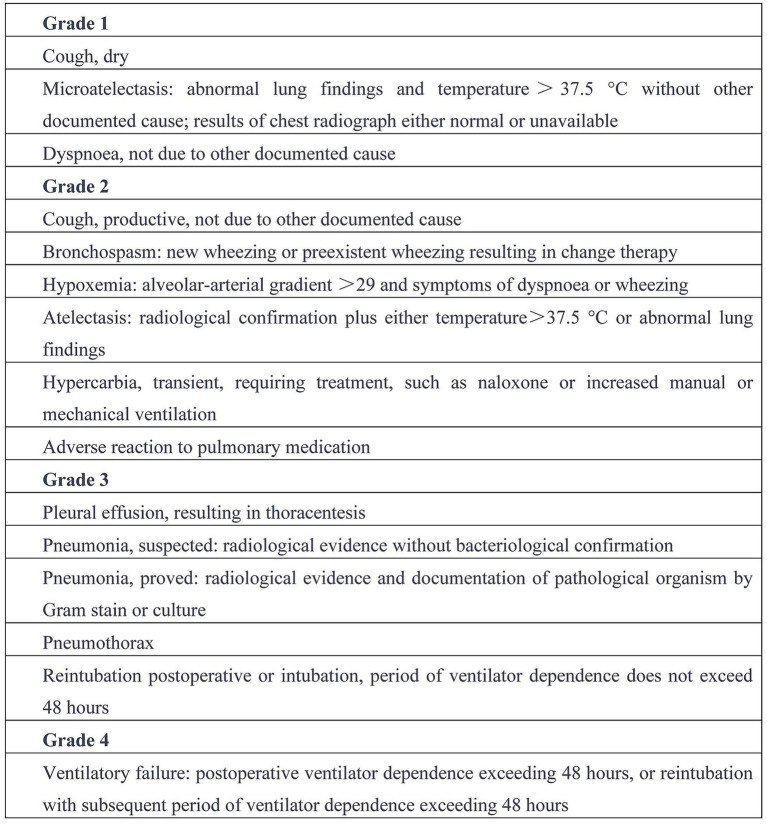
Operational definitions of postoperative pulmonary complications (23).

Measurements of PaO_2_ (partial pressure of arterial oxygen), PaCO_2_ (partial pressure of arterial carbon dioxide), the PaO_2_/FiO_2_ ratio, HR (heart rate), MAP (mean arterial pressure), and SpO_2_ (oxygen saturation) were recorded at T1, T2, T3 and T4. Peak airway pressure (Ppeak) and dynamic lung compliance (Cdyn) were directly measured on the anesthesia machine at T1, T2 and T3. The presence or absence of automatic PEEP and its value were recorded at T1. The time from the recovery of spontaneous breathing to extubation, and the total duration of their stay in the PACU, were both recorded. An independent investigator, blinded to the study details, evaluated the incidence of pulmonary complications within 7 days after surgery. These complications were scored based on the operational definitions of postoperative pulmonary complications provided by Hulzebos et al. (23) (Figure 2).

### 2.8 Safety of the intervention

The study evaluated the likelihood of potential side effects, such as transient hypotension (defined as less than 80% of baseline blood pressure) and oxygen desaturation (SpO_2_ below 95%), following regular alveolar recruitment maneuvers.

### 2.9 Statistical analysis

Unless otherwise specified, all the data are presented as the mean ± standard deviation or median (interquartile range). The Shapiro–Wilk test was used to assess the normality of the distribution. Outcomes were assessed using independent t tests, Mann–Whitney U tests and chi–square tests. A two-tailed *p* value <0.05 was considered to indicate statistical significance. Repeated-measures ANOVA was used to detect differences in the measured parameters between treatment groups and over time via a mixed-model procedure, with Bonferroni’s test for multiple comparisions. *p* values <0.05 were considered to indicate statistical significance. Data analysis was performed using SPSS software (version 25.0; SPSS, Inc., Chicago, Illinois, USA) and data visualization were carried out with GraphPad Prims 9 (GraphPad Software, SanDiego, CA, USA).

### 2.10 Sample size calculation

We used PASS 15.0 to calculate the sample size, basing our calculations on data from prior research. One study reported that lung ultrasound had an accuracy of 88% for detecting pulmonary atelectasis (9). According to our pilot study, the incidence of ultrasound-detected pulmonary atelectasis was 70%. We assumed this incidence would be halved by lung recruitment maneuvers and PEEP (15). With an alpha error of 0.05 and a power of 80%, the required sample size was calculated to be 29 patients per group. Accounting for a 20% dropout rate, we determined that a total of 73 patients would be needed, and thus we planned to enroll 80 patients in the study.

## 3 Results

**Figure 3:**
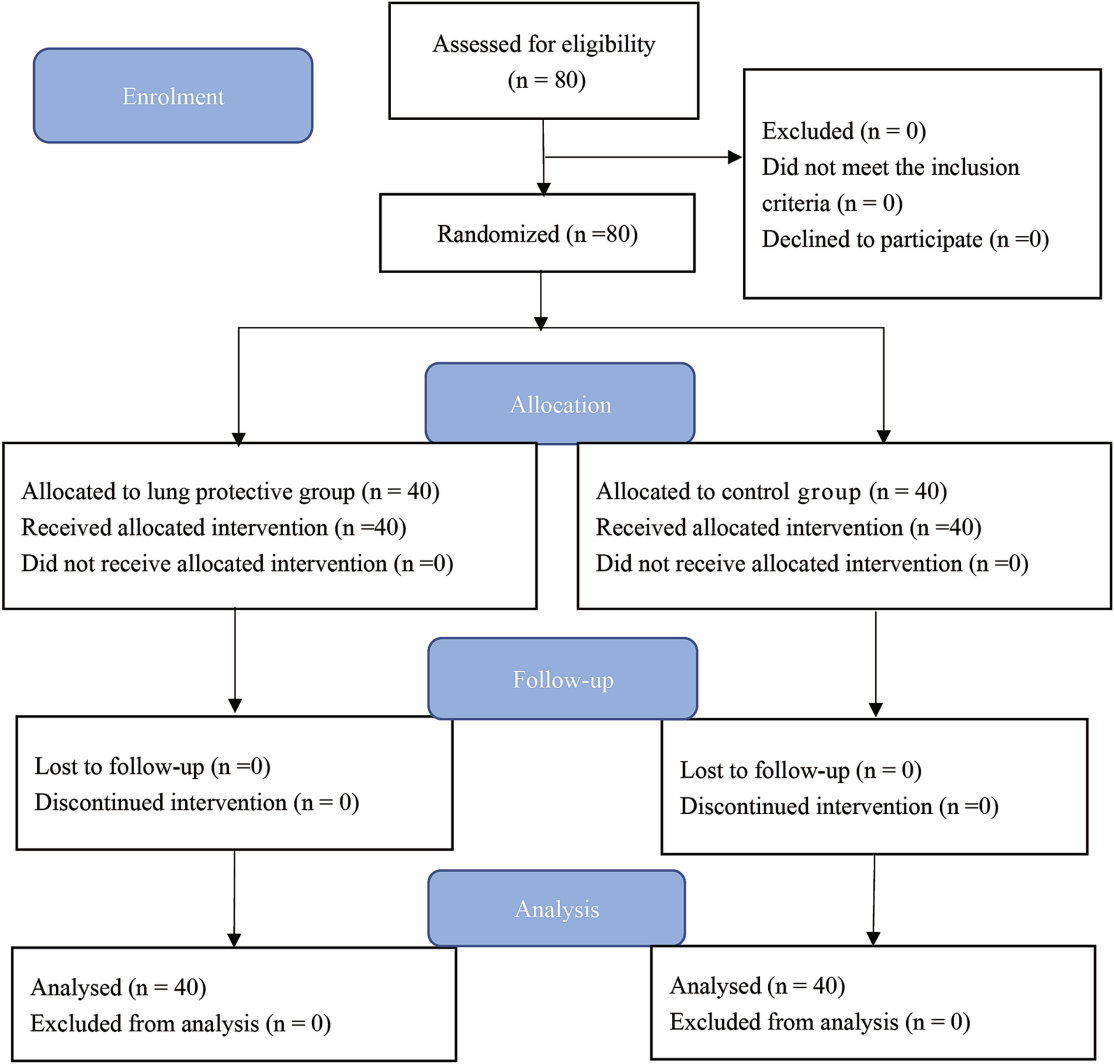
CONSORT study flow diagram.

Patient enrolment started on April 2022. A total of 80 patients were randomly assigned to the LPV (*n* = 40) or control (*n* = 40) group (Figure 3). There were no statistically significant differences in age, gender, height, weight, duration of surgery, or duration of anesthesia between the two groups of children (Table 1).

**Table 1:** Comparisons of baseline characteristics between the two groups.

Variables	LPV group (*n* = 40)	Control group (*n* = 40)	*P* value
Age/month	2.5 (1.8,4.5)	2.9 (1.7,4.4)	0.788
Sex (male/female), *n*	28/12	34/6	0.181
Height, cm	59.0 (56.5,65.0)	58.0 (55.0,64.0)	0.451
Weight, kg	6.0 (5.3,7.0)	6.1 (5.0,7.8)	0.496
Duration of surgery, min	141.5 (120.5.189.3)	153.5 (123.0,195.5)	0.630
Duration of anesthesia, min	189.5 (172.2,237.0)	189.5 (157.3,227.8)	0.507

Values are median (inter-quartile range), *n* (%).

### 3.1 Primary outcome

**Figure 4:**
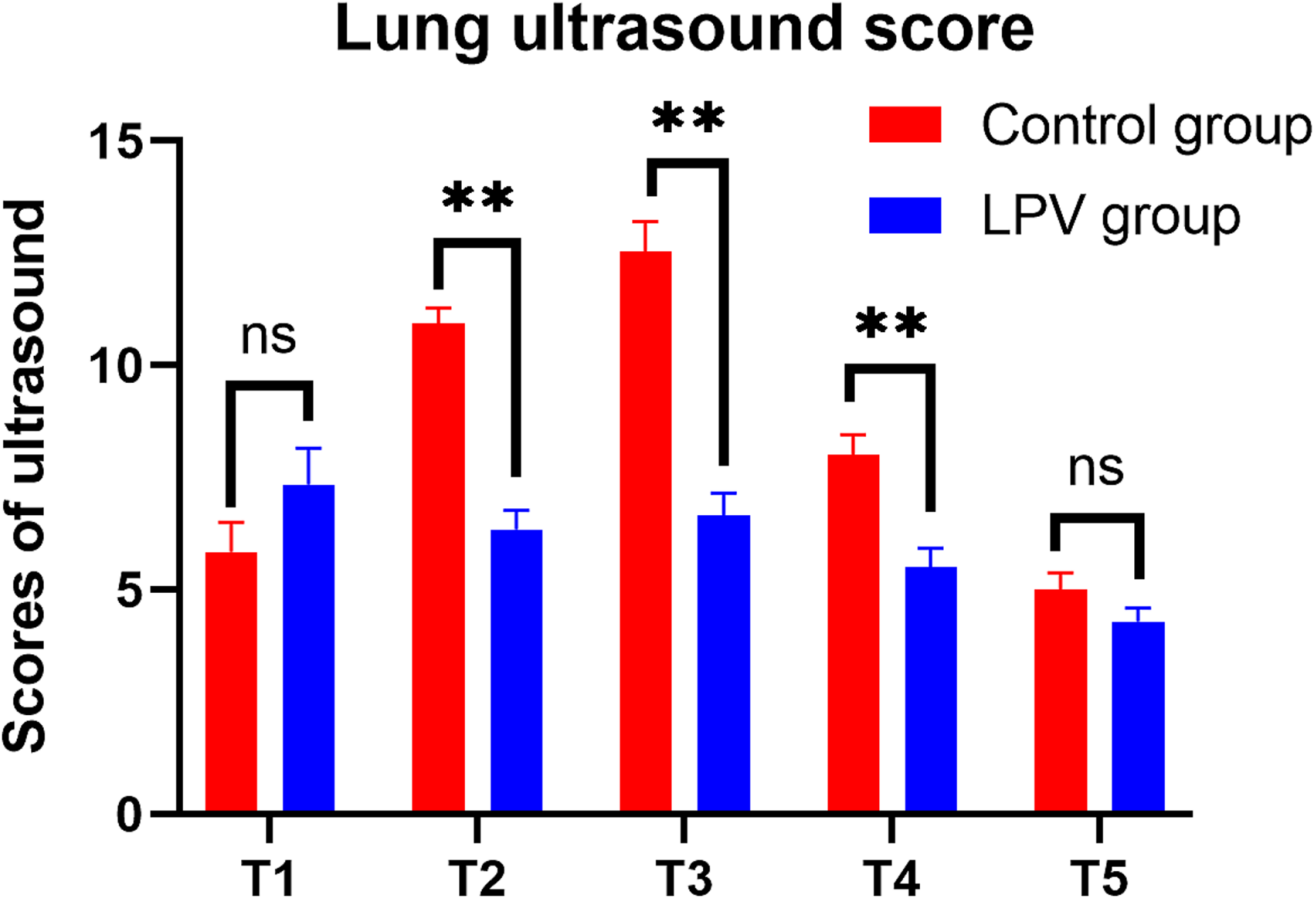
Comparison of lung ultrasound scores at different time points between the two groups. Ns, no significance. ** indicates statistical significance.

There were statistically significant differences between the LPV group and the control group at T2 in terms of ultrasound scores (6.35 ± 2.66 vs. 10.95 ± 4.07, 95% CI (−6.13 to −3.07), *p* < 0.001) and the incidence of atelectasis (62.5% vs. 87.5%, *p* = 0.010); at T3 in terms of ultrasound scores (6.65 ± 3.15 vs. 12.53 ± 4.29, 95% CI (−7.55 to −4.20), *p* < 0.001) and the incidence of atelectasis (55% vs. 90%, *p* = 0.0005); and at T4 in terms of ultrasound scores (5.50 ± 2.75 vs. 8.00 ± 2.92, 95% CI (−3.76 to −1.24), *p* < 0.001) and the incidence of atelectasis (35% vs. 72.5%, *p* = 0.001). At T1, there was no statistically significant difference in lung ultrasound score (7.35 ± 5.06 vs. 5.85 ± 4.14, 95% CI (−0.56 to 3.56), *p* = 0.151), the incidence of atelectasis (80% vs. 77.5%, *p* = 0.785). Moreover, there were no statistically significant differences between the LPV group and the control group at T5 in terms of ultrasound score (4.30 ± 1.87 vs. 5.00 ± 2.43, 95% CI (−1.67 to 0.27), *p* = 0.153) or incidence of atelectasis (32.5% vs. 47.5%, *p* = 0.171) (Table 2). Ultrasound scores of both groups at various time points are presented in Figure 4.

**Table 2:** Lung ultrasound score and comparison of intra- and postoperative variables between the LPV group and control group.

	Parameters	LPV group (*n* = 40)	Control group (*n* = 40)	Mean differences (95% CI)	*P* value
T1	Incidence of significant atelectasis	32 (80)	31 (77.5)		0.785
	Lung ultrasound score	7.35 ± 5.06	5.85 ± 4.14	1.50 (−0.56 to 3.56)	0.151
	PaO_2_ (mmHg)	260.64 ± 23.43	262.39 ± 25.89	−1.75 (−12.74 to 9.25)	0.752
	PaCO_2_ (mmHg)	38.32 ± 3.15	37.94 ± 4.16	0.37 (−1.27 to 2.02)	0.653
	PaO_2_ ∕FiO_2_ (mmHg)	434.40 ± 39.05	437.31 ± 43.16	−2.91 (−21.23 to 15.41)	0.752
	P_peak_ (cmH_2_O)	11.0 (10.3,12.0)	14 (13.0,15.0)		<0.001*
	Cydn (mL/cmH_2_O)	6.34 ± 2.44	4.17 ± 1.10	2.17 (1.33 to 3.00)	<0.001*
	autoPEEP (cmH_2_O)	0 (0,1)	0 (0,1)		0.772
T2	Incidence of significant atelectasis	25 (62.5)	35 (87.5)		0.010*
	Lung ultrasound score	6.35 ± 2.66	10.95 ± 4.07	−4.60 (−6.13 to −3.07)	<0.001*
	PaO_2_ (mmHg)	248.26 ± 24.30	209.62 ± 29.68	38.64 (26.57 to 50.72)	<0.001*
	PaCO_2_ (mmHg)	47.68 ± 3.36	38.34 ± 2.86	9.35 (7.96 to 10.73)	<0.001*
	PaO_2_∕FiO_2_ (mmHg)	413.77 ± 40.50	349.37 ± 49.47	64.40 (44.28 to 84.53)	<0.001*
	P_peak_ (cmH_2_O)	14.0 (14.0,15.0)	17 (17.0,18.0)		<0.001*
	Cydn (mL/cmH_2_O)	4.07 ± 1.26	3.39 ± 0.89	0.68 (0.19 to 1.17)	<0.001*
T3	Incidence of significant atelectasis	22 (55)	36 (90)		0.0005*
	Lung ultrasound score	6.65 ± 3.15	12.53 ± 4.29	−5.88 (−7.55 to −4.20)	<0.001*
	PaO_2_ (mmHg)	246.94 ± 25.49	172.53 ± 14.86	74.41 (65.12 to 83.70)	<0.001*
	PaCO_2_ (mmHg)	48.24 ± 3.39	40.99 ± 2.35	7.26 (5.96 to 8.56)	<0.001*
	PaO_2_ ∕FiO_2_ (mmHg)	411.56 ± 42.49	287.54 ± 24.76	124.02 (108.54 to 139.50)	<0.001*
	P_peak_ (cmH_2_O)	11.0 (11.0,12.0)	14.5 (13.0,15.0)		<0.001*
	Cydn (mL/cmH_2_O)	5.93 ± 1.93	4.10 ± 1.07	1.83 (1.14 to 2.53)	<0.001*
T4	Incidence of significant atelectasis	14 (35)	29 (72.5)		0.001*
	Lung ultrasound score	5.50 ± 2.75	8.00 ± 2.92	−2.50 (−3.76 to −1.24)	<0.001*
	PaO_2_ (mmHg)	85.42 ± 3.73	72.04 ± 6.72	13.38 (10.96 to 15.80)	<0.001*
	PaCO_2_ (mmHg)	40.65 ± 3.58	40.55 ± 3.21	0.10 (−1.41 to 1.62)	0.893
	PaO_2_ ∕FiO_2_ (mmHg)	427.09 ± 18.63	360.20 ± 33.59	66.89 (54.80 to 78.98)	<0.001*
T5	Incidence of significant atelectasis	13 (32.5)	19 (47.5)		0.171
	Lung ultrasound score	4.30 ± 1.87	5.00 ± 2.43	−0.70 (−1.67 to 0.27)	0.153
Time to extubation, min		15.0 (13.0,18.5)	18.0 (16.0,20.0)		0.003*
PACU duration, min		29.5 (29,33)	34.0 (30.5,35.8)		<0.001*

Values are median (inter-quartile range), *n* (%), or mean ± standard deviation. * *P* value <0.05. T1 was 5 min post-intubation, T2 was 5 min post-pneumoperitoneum, T3 was at the end of surgery, T4 was post-extubation, and T5 was prior to discharge from the PACU.

### 3.2 Secondary outcomes

At T1, there was no statistically significant difference in PaO_2_ (260.64 ± 23.43 vs. 262.39 ± 25.89, 95% CI (−12.74 to 9.25), *p* = 0.752), PaCO_2_ (38.32 ± 3.15 vs. 37.94 ± 4.16, 95% CI (−1.27 to 2.02), *p* = 0.653), or the PaO_2_/FiO_2_ ratio (434.40 ± 39.05 vs. 437.31 ± 43.16, 95% CI (−21.23 to 15.41), *p* = 0.752). There were statistically significant differences between the LPV group and the control group at T2 and T3 in terms of the PaO_2_, PaCO_2_, PaO_2_/FiO_2_ or Cdyn; at T4, in terms of the PaO_2_ or PaO_2_/FiO_2_. The LPV group demonstrated shorter extubation times and reduced lengths of stay in the PACUcompared to the control group (Table 2). There was no significant difference in autoPEEP between the two groups at T1. The two groups showed no significant difference in HR, MAP or SpO_2_ at T1, T2, T3, or T4 (Table 3). No significant difference was observed in the incidence of pulmonary complications within 7 days after surgery (*p* = 0.516) (Table 4). Alveolar recruitment maneuvers did not show transient hypotension and oxygen desaturation.

**Table 3:** Changes in vital signs at each time point between the LPV group and the control group.

	LPV group (*n* = 40)	Control group (*n* = 40)	*P* value
MAP (mmHg) at T1	55.83 ± 5.78	57.05 ± 6.40	0.300
HR (bpm) at T1	130.43 ± 9.74	128.54 ± 9.67	0.434
SpO_2_ (%) at T1	100 (97–100)	100 (98–100)	0.937
MAP (mmHg) at T2	56.25 ± 4.05	57.49 ± 4.20	0.148
HR (bpm) at T2	123.25 ± 6.34	121.18 ± 6.73	0.179
SpO_2_ (%) at T2	100 (97–100)	100 (98–100)	0.706
MAP (mmHg) at T3	55.60 ± 4.04	56.28 ± 3.83	0.368
HR (bpm) at T3	118.95 ± 5.03	117.56 ± 4.57	0.219
SpO_2_ (%) at T3	100 (98–100)	100 (98–100)	0.513
MAP (mmHg) at T4	60.75 ± 3.41	61.77 ± 4.21	0.240
HR (bpm) at T4	132.93 ± 9.29	132.97 ± 8.79	0.981
SpO_2_ (%) at T4	100 (97–100)	100 (96–100)	0.259

Values are mean ± standard deviation, median (range).

**Table 4:** Incidence of PPCs within 7 days after surgery [*n* (%)].

Groups	*n*	Incidence of PPCs (*n*, %)	Grading of PPCs			
			Grade 1	Grade 2	Grade 3	Grade 4
LPV group*	40	4 (10)	3	1	0	0
Control group	40	7 (17.5)	5	2	0	0

PPCs, Postoperative Pulmonary Complications.* The Incidence of PPCs within 7 days after surgery did not differ between the groups, *p* = 0.516.

## 4 Discussion

In this prospective RCT, the primary outcome indicated that lung protective ventilation reduced the incidence of pulmonary atelectasis in infants during laparoscopy under general anesthesia compared to conventional ventilation, enhanced intraoperative oxygenation and Cdyn, the duration of extubation and the length of stay in the PACU were both reduced. However, this improvement was transient, with no observed differences in lung ultrasound scores or incidence of pulmonary atelectasis at discharge from the PACU. Moreover, no significant differences were observed in the incidence of postoperative pulmonary complications within 7 days after surgery.

Our results align with previous studies (7, 15) demonstrating that laparoscopic surgery under general anesthesia can lead to pulmonary atelectasis in children, we provide data on infants. Acosta et al. (15) discovered that lung collapse resulting from capnoperitoneum can be mitigated through the use of LPV in all children aged 6 months to 7 years undergoing laparoscopic surgery. The incidence of pulmonary atelectasis resulting from pneumoperitoneum can potentially be reduced through the use of LPV. The primary mechanisms involve the application of lung recruitment maneuvers and PEEP, which effectively mitigate diaphragmatic elevation and subsequent intra-abdominal pressure increase caused by intraperitoneal gas accumulation (24). Recruitment maneuvers can re-expand atelectatic lung tissue and improve lung compliance; however, they may also carry risks such as barotrauma, hemodynamic instability, and worsening oxygenation (14), the pressure-volume curve or loop method, the end-expiratory lung volume–static compliance method, and ultrasound can all be used to assess the effectiveness of lung recruitment (7, 25). Other studies have shown that the optimal PEEP under RM can be determined based on the static compliance of the respiratory system (crs) (26). This study revealed that combining lung recruitment maneuvers with PEEP effectively reopened collapsed alveoli and enhanced intraoperative oxygenation and dynamic lung compliance during laparoscopic surgery in small infants. However, one limitation is that the extent of alveolar recruitment remains somewhat unclear.

Our findings indicate that recruitment maneuvers, followed by a PEEP of 5 cmH_2_O, are both effective and safe for reducing the number of atelectatic areas following laparoscopic surgery in young infants, but the specific numerical setting for PEEP remains controversial. J-H Lee’s (4) study posits that a PEEP of 10 cm H₂O is an appropriate level for children in mechanically ventilation. However, other studies (6, 27) have indicated improvements in lung ultrasound scores and reductions in the incidence of atelectasis in children administered a PEEP of 5 cmH₂O. Although the application of PEEP facilitates the reopening of collapsed alveoli and improves oxygenation, the use of high levels of PEEP must be carefully considered due to potential risks such as alveolar overdistension and hemodynamic instability (28). These effects are particularly undesirable in infants undergoing laparoscopic surgery. Due to potential side effects, clinicians must exercise caution when using 10 cmH_2_O PEEP during laparoscopic surgery for small infants. For safety considerations, this study employed 5 cmH₂O PEEP in the LPV group.

Studies have shown that the detrimental effects of capnoperitoneum could be reversed by a protective ventilation strategy combining lung recruitment and an individualized PEEP titration during laparoscopic surgery in children (5), the individualized lung protective ventilation during laparoscopic surgery in young infantsshould be the matter of future studies.

Avoiding high fractions of oxygen in inspired gas during induction and maintenance of anesthesia may prevent the formation of atelectasis (29). Studies have shown that a lower oxygen concentration during anesthesia induction is associated with a lower risk of atelectasis immediately after anesthesia induction in children, and 60% oxygen should be applied to prevent atelectasis (11). The changes in oxygen concentration were consistent in both groups, with 60% FiO_2_ used at induction and intraoperatively, potentially reducing the occurrence of pulmonary atelectasis. At extubation, the air oxygen concentration was used, and in the PACU, the infants’ FiO_2_ was 0.29. To avoid residual effects of anesthetic drugs, incomplete lung re-expansion, reduced chest wall, and diaphragmatic activity caused by surgical injury and pain, and gastrointestinal reactions (30), postoperative oxygen respiratory support was maintained during the awakening period until SpO_2_ stabilized at 95% or higher. Once the SpO_2_ level exceeded 95% and stabilized, the FiO_2_ was gradually reduced to room air levels. The infants in both groups did not develop hypoxemia after breathing air. The potential effects of oxygen concentration changes warrant further exploration.

Our study demonstrated that patients in both groups experienced significant improvements in pulmonary ventilation after extubation, despite variations in PaO_2_, extubation times, and lengths of stay in the PACU. There were no statistically significant differences in ultrasound scores and incidence of significant pulmonary atelectasis in infants discharged from the PACU, which is consistent with the findings of Zhu’s (18) study. Some patients might have performed uncontrolled recruitment maneuvers by sighing or coughing (31), the sigh is a normal homeostatic reflex that maintains lung compliance and decreases the risk of atelectasis (32). Both groups of infants have different body movements, coughing or crying, which may improve ventilation of the lungs.

Despite hypercapnia occurring during the intraoperative period, it was restored to acceptable levels by the time of extubation (33). The findings of our research indicate that LPV not only enhances ventilation and mitigates intraoperative atelectasis but also preserves hemodynamic stability without inducing fluctuations. Our secondary findings indicated no variation in respiratory complications within 7 days after surgery, which may relate to the normal respiratory physiology of the infants included in the study. In fact, the potential of different tidal volumes to reduce the risk of postoperative pulmonary complications depends on the patient’s respiratory compliance (34). Moreover, large-sample, multicenter studies are necessary to further investigate the clinical significance of lung protective ventilation in small infant laparoscopic surgery.

Our study has several limitations. First, we included only infants with normal respiratory physiology, and those with lung disease or who were critically ill were not evaluated. Second, while all sonographers involved in our study were professionally trained, the observed atelectasis may be explained by the fact that small atelectasis can be hidden within the rib’s acoustic shadows whenever the longitudinally oriented probe placed in the traditional orientation crosses the rib (9), during the second ultrasound examination, factors such as the patient’s positioning, the presence of surgical drapes, the requirement for sterility, and the surgeon’s maneuvers can impede the accessibility and effectiveness of the ultrasound, we conducted a comprehensive examination of pulmonary ultrasound images by adjusting the probe orientation and pausing the surgery to minimize the potential for misinterpretation (35). Third, we were unable to confirm the effect of recruitment maneuvers, although we assessed the potential risks associated with recruitment maneuvers and adhered to the established standards for their implementation, we could not rule out the possibility of overinflation, and we applied a PEEP of 5 cmH_2_O in this trial, which might not be the optimal PEEP during laparoscopic surgery in infants. Fourth, we did not take into account the effect of autoPEEP when setting up ventilation, we recorded auto PEEP during mechanical ventilation after endotracheal intubation, however, the data of the two groups showed no difference, equalizing the possible influence of auto PEEP on the results. And we looked for the etiology and manage patients with auto PEEP (17), such as the use of increased depth of anesthesia to reduce sputum obstruction and avoid airway spasm, as well as the avoidance of thinner tracheal intubation (36, 37), thus reducing the potential impact that autoPEEP could have on this study. Further exploration of individualized PEEP in small infants via laparoscopy at a later date is warranted.

In conclusion, lung protective ventilation significantly reduced the incidence of pulmonary atelectasis in infants aged 1–6 months during laparoscopy under general anesthesia compared with conventional ventilation and improved intraoperative oxygenation and Cdyn, reduced in both the time to extubation and the length of stay in the PACU, however, pulmonary atelectasis did not seem to last very long, there was no difference in its incidence at discharge from the PACU, and nor in postoperative pulmonary complications within 7 days after surgery.

## Data Availability

The raw data supporting the conclusions of this article will be made available by the authors, without undue reservation.
